# Overlap of immunotherapy-related pneumonitis and COVID-19 pneumonia: diagnostic and vaccine considerations

**DOI:** 10.1136/jitc-2020-002307

**Published:** 2021-04-30

**Authors:** Muhammad Bilal Abid

**Affiliations:** Divisions of Hematology/Oncology & Infectious Diseases, Department of Medicine, Medical College of Wisconsin, Milwaukee, Wisconsin, USA

**Keywords:** COVID-19, immunity, cellular, immunotherapy, vaccination, autoimmunity

## Abstract

The clinically indistinguishable overlap between pneumonitis caused due to immune checkpoint inhibition (ICI) and pneumonia associated with COVID-19 has posed considerable challenges for patients with cancer and oncologists alike. The cancer community continues to face the challenges that lay at the complex immunological intersection of immune-based cancer therapy and immune dysregulation that results from COVID-19. Is there compounded immune dysregulation that could lead to poor outcomes? Could ICIs, in fact, ameliorate SARS-CoV-2-driven T-cell exhaustion?

A little more is known about the kinetics of the viral replication in immunocompromised patients now as compared with earlier during the pandemic. Working knowledge of the diagnostic and therapeutic nuances of SARS-CoV-2 infection in patients with active cancers, issues related to viability and replication potential of the virus, unclear role of corticosteroids among those with diminished or dysfunctional effector T-cell repertoire, and the type of immunotherapy with differential risk of pneumonitis will inform decision making related to immunotherapy choices and decision for ICI continuation in the era of COVID-19.

The clinically indistinguishable overlap between pneumonitis caused by immune checkpoint inhibition (ICI) and pneumonia associated with COVID-19 has posed considerable challenges for patients with cancer and oncologists alike. The cancer community continues to face the challenges that lay at the complex immunological intersection of immune-based cancer therapy and immune dysregulation that results from COVID-19.^1^ Is there compounded immune dysregulation that could lead to poor outcomes? Or if ICIs, in fact, could ameliorate SARS-CoV-2-driven T-cell exhaustion? The positive association between ICI-mediated immune-related adverse events (irAEs) and survival adds further fuel to the fire.^2^

The recent rise in COVID-19 cases globally, referred to as the second wave, along with the associated challenges, also brings in opportunities for improved care for patients undergoing ICIs. A little more is known about the kinetics of the viral replication in immunocompromised patients now as compared with earlier during the pandemic. Working knowledge of the diagnostic and therapeutic nuances of SARS-CoV-2 infection in patients with active cancers, issues related to the viability and replication potential of the virus, unclear role of corticosteroids among those with diminished or dysfunctional effector T-cell repertoire and the type of immunotherapy with differential risk of pneumonitis will inform decision making related to immunotherapy choices and decision for ICI continuation in the era of COVID-19.

Cancer cells evade the host’s immunity by inhibiting immune effector cells, particularly cytotoxic (CD8+) T cells, by employing immune checkpoint pathways, such as programmed cell death-1 (PD-1) and cytotoxic T-lymphocyte antigen-4 (CTLA-4). The ligands for PD-1, programmed cell death ligand 1 (PD-L1) and programmed cell death ligand 2‚ as well as the ligands for CTLA-4, B7-1 (CD80), and B7-2 (CD86), are upregulated in both solid tumors and hematological malignancies. Two PD-1 inhibitors (nivolumab and pembrolizumab), three PD-L1 inhibitors (atezolizumab, durvalumab, and avelumab), and one CTLA-4 inhibitor (ipilimumab) have been approved by the US Food and Drug Administration (FDA) for a myriad of cancers.^3^ With the ever-increasing use of ICIs across cancers, both in the frontline and relapsed setting, the incidence of ICI-related pneumonitis is likely higher than reported in the existing literature.

Theoretically, ICIs are assumed to have an immunostimulatory effect—unleashing effector T cells from checkpoint-mediated immunosuppressive tumor microenvironment and, hence, potentially leading to autoimmunity. However, the actual directional impact of ICIs on the immune system is unknown. In the real world, while ICIs have been used to treat chronic viral infections (such as pembrolizumab for progressive multifocal leukoencephalopathy (PML), caused by John Cunningham (JC) virus), there have also been reports that ICIs have led to the reactivation of latent tuberculosis and Epstein-Barr Virus (EBV)-driven idiosyncratic irAE.^4^ Phenotypical similarities in the patterns of T-cell exhaustion are likely the common denominator resulting in both chronic viral infections (hepatitis B virus, hepatitis C virus, HIV, and PML due to JC virus) as well as tumor-related immune evasion.

While ICI-related pneumonitis accounts for up to a third of all deaths caused by irAE, its risk and severity are not uniformly distributed. Pulmonary toxicity occurs at varying rates, and incidence and severity depend on the underlying tumor type, pre-existing pulmonary architecture, the type of ICI, the dosage and frequency, and whether the ICIs are used as monotherapy versus in combination (table 1).

**Table 1:** Patient, disease, and COVID-19 related factors suggested for consideration for immunotherapy

Suggestions/considerations	Additional notes
**Disease/Tumor-related**	
Tumor type	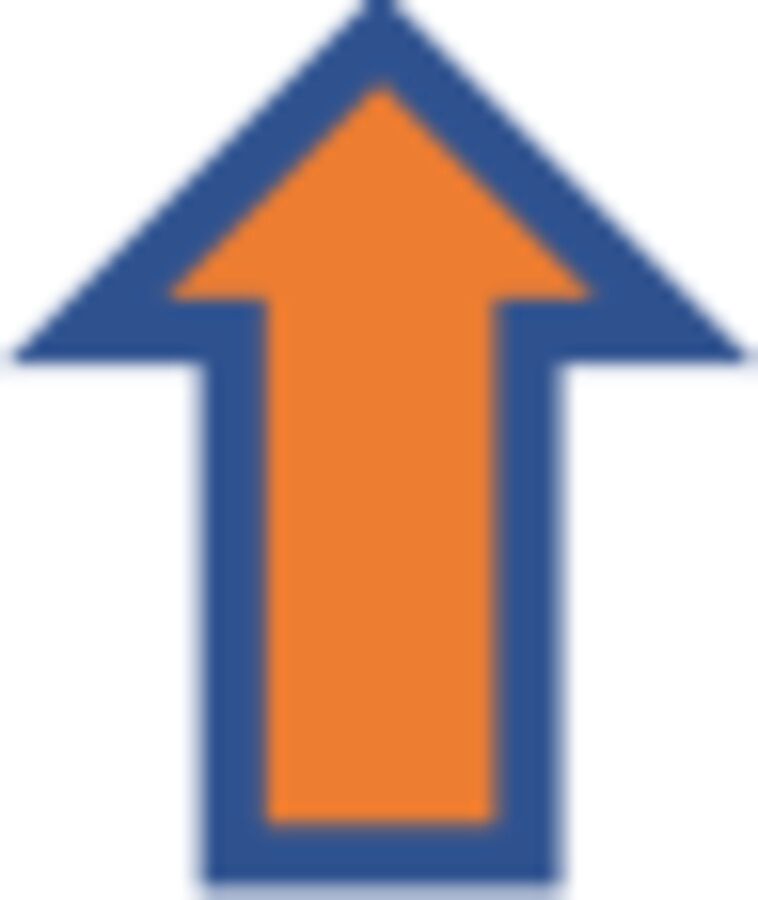 risk with NSCLC as compared with RCC and melanoma
Tumor histology	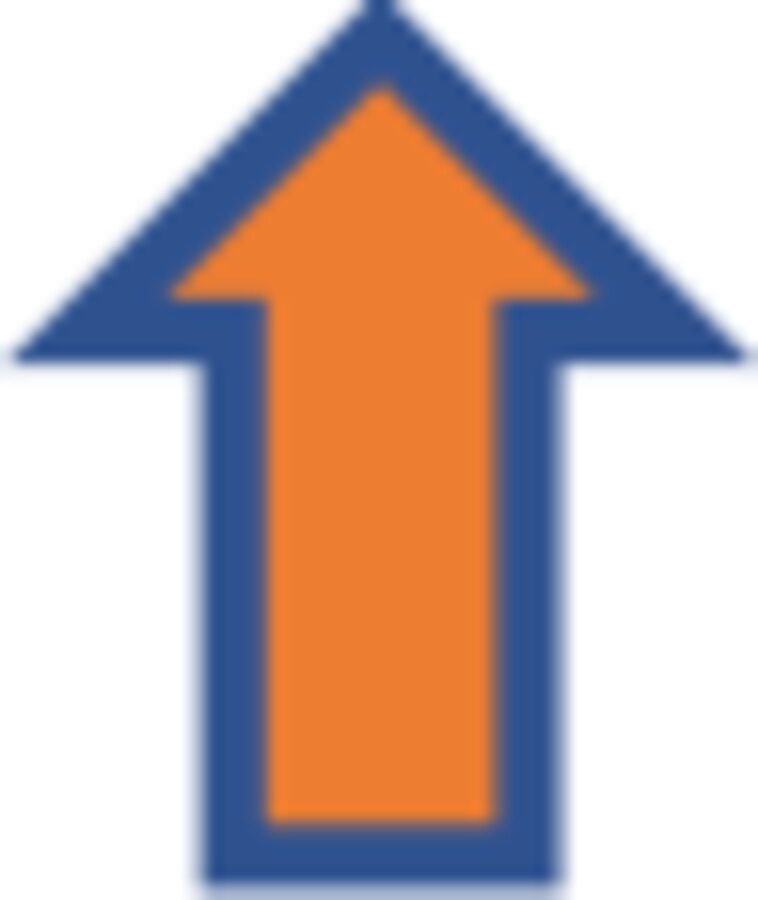 risk with non-adenocarcinoma (including squamous NSCLC) as compared with adenocarcinomas
Disease status	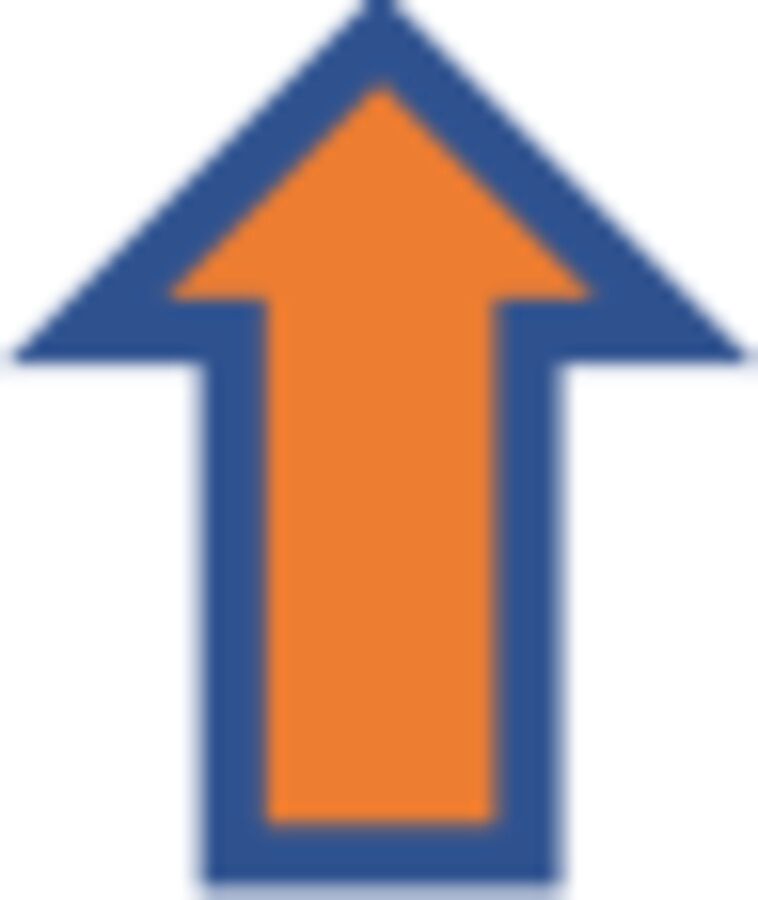 risk with progressive / refractory disease
Burden of disease	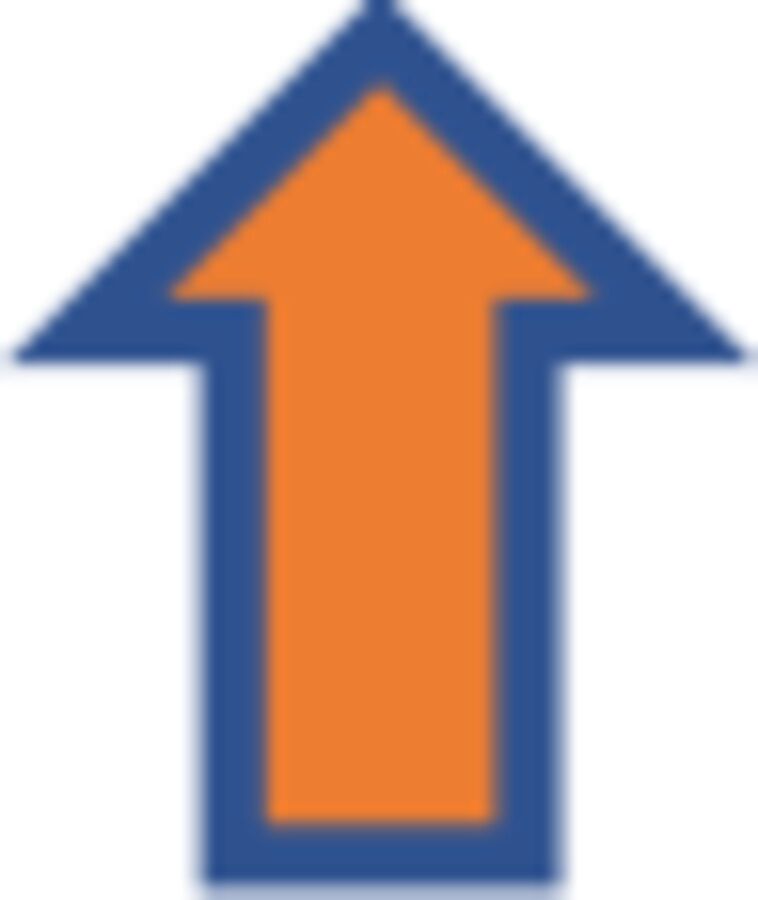 risk with bulky / metastatic disease
Prior receipt of chest radiotherapy	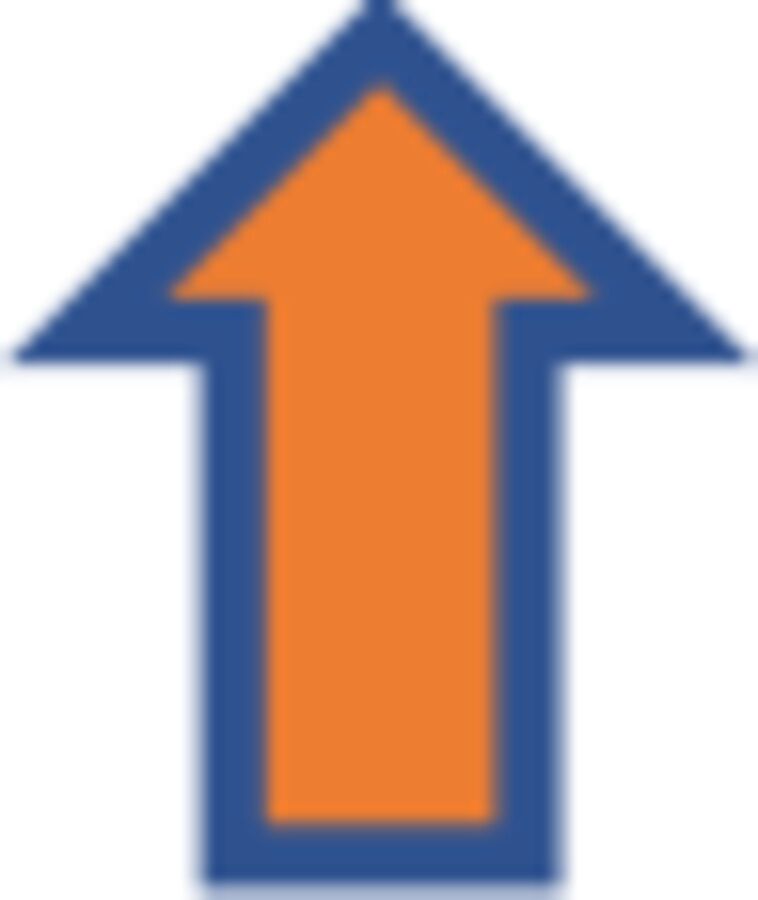 risk with history of prior radiotherapy
Genetic markers	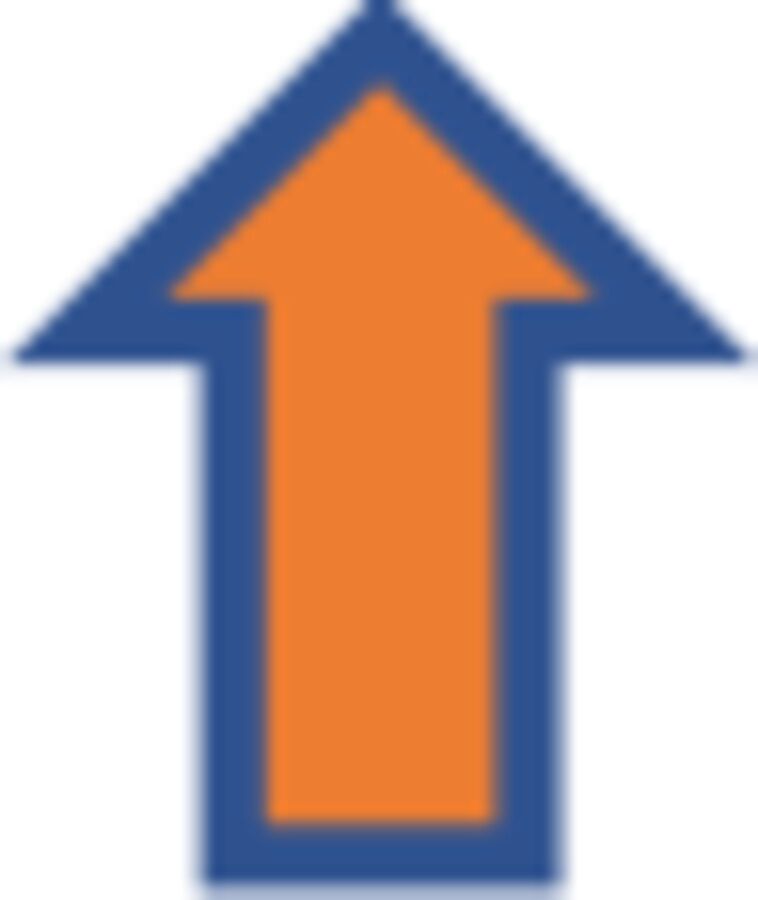 risk in patients with NSCLC with *EGFR* mutation. Consider if alternative therapies available
	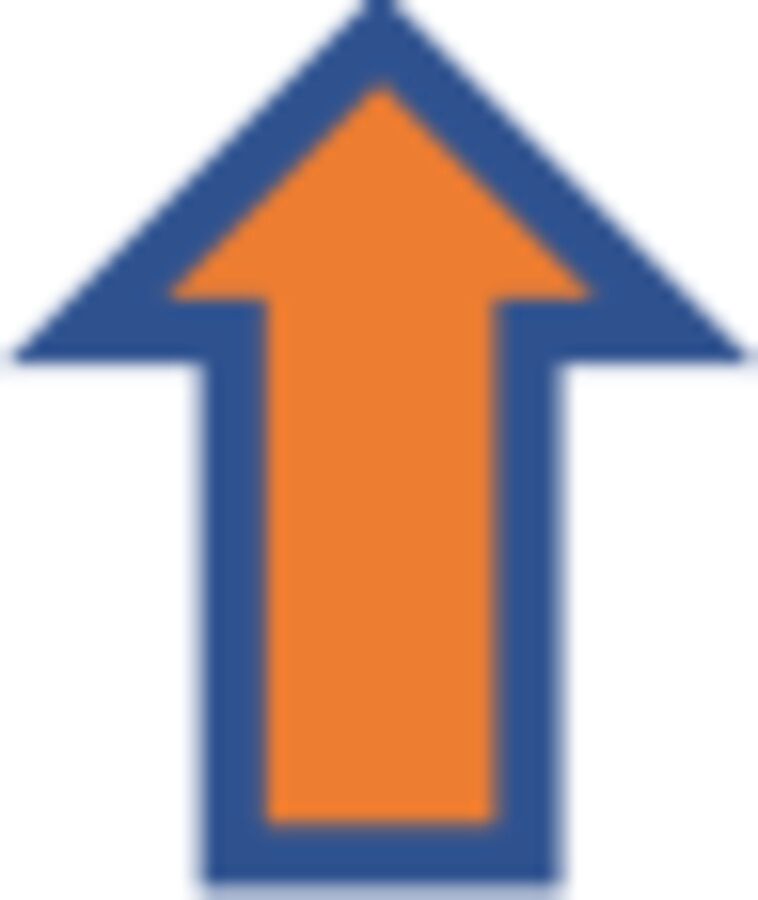 risk of HPD with *MDM/EGFR* gene mutations
**Immunotherapy-related**	
Indication (disease/setting)	
Intent of treatment	Curative potential, alternative treatment options
Combination vs monotherapy	Twice as much risk with combination immunotherapy regimen
Risk of immunotherapy -related pneumonitis	PD-1i > CTLA-4 or PD-L1i; Among PD-1i (pembrolizumab > nivolumab)
Consider FDA-approved four weekly nivolumab for select indications and six weekly pembrolizumab for all indications	Monitor closely in the initial post-immunotherapy period for harsher irAE
**Infection-related**	
Recent travel, exposures	
Ongoing symptom review	
Baseline chest CT	Whenever applicable
Prevalence of COVID-19 in the area	
Baseline (pre-immunotherapy) CD4+	Whenever applicable
Baseline (pre-immunotherapy) IgG	Whenever applicable
Two COVID-19 NP PCR swab negative (at least 48 hours apart), prior to initiation	
Ct value > 30 (and uptrending) among those with prior COVID-19	Caution advised when applying published correlations of Ct values with disease severity or as a predictor of active infection and transmissibility
Weekly surveillance COVID-19 PCR testing while on immunotherapy	
**Patient-related**	
Age	Higher risk in the elderly
Performance status	
Comorbidities	
Underlying pulmonary architecture	Asthma, COPD
Smoking status	Non-smoker>prior smoker>active

COPD, chronic obstructive pulmonary disease; COVID-19, Coronavirus Disease 2019; CT, computed tomography; Ct, Cycle threshold; CTLA-4, cytotoxic T-lymphocyte antigen-4; EGFR, epidermal growth factor receptor; HPD, hyperprogressive disease; IgG, immunoglobulin G; irAE, immune-related adverse events; NP, nasopharyngeal; NSCLC, non-small cell lung carcinoma; PCR, polymerase chain reaction; PD-1i, programmed cell death–1 inhibitor; PD-L1i, programmed cell death ligand 1 inhibitor; RCC, renal cell carcinoma.

The severity of ICI-related pneumonitis is graded per the Common Terminology Criteria for Adverse Events V.4.03 and the management of respective grades are outlined elsewhere.^5^ A meta-analysis that included data from 20 clinical trials and nearly 4500 patients with solid tumors showed that the incidence of ICI-related pneumonitis was 2.7% and up to 4% of patients discontinued ICIs due to pulmonary toxicity.^6^ The risk is considerably higher with PD-1 inhibitors as compared with CTLA-4 or PD-L1 inhibitors. Among Food and Drug Administration (FDA)-approved PD-1 inhibitors, the risk of pneumonitis is reportedly higher with pembrolizumab as compared with nivolumab. The risk further increases with combination versus monotherapy, approaching as much as three times when given in combination. Furthermore, the risk of developing ICI-related pneumonitis varies with tumor type and histology. The incidence is reported to be higher with non-small cell lung carcinoma (NSCLC) and renal cell carcinoma as compared with melanoma and with non-adenocarcinoma (including squamous NSCLC) as compared with adenocarcinomas tumor histology. Real-world data also suggest that patients with NSCLC are at a particularly higher risk of developing ICI-related pneumonitis. The incidence in this cohort of patients is reportedly as high as 19%.^7^

Additionally, other clinical factors that ought to be considered when deciding on initiation or continuation of ICIs for patients who have recovered from COVID-19 or who are at risk of exposure or those who reside in areas with moderate to high community spread of COVID-19. Smoking is shown to be associated with an increased risk of ICI-related pneumonitis.^6 8 9^ While male gender has been signaled as a risk factor, a similar association with age has not been demonstrated consistently.^6 9^ Other factors for the development of ICI-related pneumonitis include underlying lung conditions (asthma and chronic obstructive pulmonary disease), smoking status (non-smoker vs active/prior smoker), NSCLC with *EGFR* mutation, prior receipt of chest radiotherapy, or history of treatment with tyrosine kinase inhibitors in combination with ICIs.^7 9 10^ Additional considerations for genetic mutation predisposing to hyperprogressive disease, such as *MDM* family and *EGFR*, may also be considered prior to the initiation of ICIs. The duration of onset of pneumonitis due to ICIs further varies and, again, depends on the ICI regimen and tumor type. Pneumonitis is more likely to develop earlier with NSCLC than with melanoma and with combination ICIs than with single-agent.^11^ Furthermore, earlier-onset pneumonitis is likely to be of higher grade (severe) as compared with pneumonitis that occurs late after ICI therapy.^7^

ICI-related pneumonitis remains a diagnosis of exclusion and additional diagnostic studies must be considered. Key differential diagnoses, including infections, tumors, and other autoimmune processes, ought to be ruled out in a timely manner in order to allow prompt institution of appropriate management. In addition to the intersecting radiological features of the two disease entities, the overlapping histopathological features predominantly include diffuse alveolar damage and interstitial fibrosis.

The delay in diagnosis is further compounded by reports of false-negative upper airway testing (nasopharyngeal and oropharyngeal) and issues with sampling methods. The data, thus far, underscore a potentially distinctive viral shedding pattern in immunocompromised patients due to both an inability to contain the virus early (numerical and functional compromise in effector T cells) and an abundance of viral replication in the lower tract early (ACE2 overexpression as well as preferential viral tropism).^12^ This was further corroborated in a preclinical study in which the investigators demonstrated that mitogen-activated protein kinase inhibitors showed promise in treating SARS-CoV-2 infection not just by stimulating cells of the innate immune system and inhibiting inflammatory cytokines but also by decreasing ACE2 expression on pulmonary epithelial cells of a SARS-CoV-2-S pseudovirus as well as on a lung cancer cell line.^13 14^ While early bronchoscopic evaluation would be ideal in the case of false-negative upper airway testing (reverse transcriptase PCR assay) in patients with cancer undergoing ICI and suspected or at high risk of COVID-19, it is not practical, given the risk of exposure to the healthcare providers and proceduralists.^15^ Hence, presumptive treatment may also be considered in select cancer patients to minimize exposure to healthcare providers.

Evolving data have shown that patients with cancer undergoing active treatment have poor outcomes due to COVID-19. A recent study evaluating 3920 patients with cancer with COVID-19 showed an 18% 30-day mortality in patients who underwent immunotherapy within 1–3 months prior to COVID-19.^16^ Active chemotherapy or immunotherapy lead to protracted lymphopenia, which, in turn, results in higher viral load in patients with cancer. However, the data exclusively examining ICI remain conflicting. Given the lack of convincing evidence demonstrating immunosuppression with ICIs, this effective and survival-prolonging treatment modality should not be avoided to curb the hypothetical risk of severe COVID-19 until conclusive data become available.^17 18^ The US Food and Drug Administration’s (FDA) approval of 4-weekly nivolumab for select indications and 6-weekly pembrolizumab for all indications is timely in this regard.

Patients with cancer are at risk of prolonged shedding of replication-incompetent virus, for many months in some cases.^12 15 19^ Hence, a negative PCR may not be achievable or practical in a subset of asymptomatic patients: those with a persistently positive SARS-CoV-2 PCR and a rapidly progressive underlying cancer. In such select cases in which the patient is asymptomatic but has not had two negative PCRs 48 hours apart or cannot wait due to risk of progression of the underlying disease, an up-trending cycle threshold (Ct) value that is at least greater than 30-35 and is performed on a standardized platform, ICIs may be initiated/resumed.^20^ However, the nuances associated with Ct values must be considered. Given that qualitative RT-PCR testing generates binary results and are not designed to provide a quantitative measurement of nucleic acid in a sample, coupled with lack of conclusive data on optimal Ct values that correlate with viral culture negativity (in the setting of PCR positivity), additional data are needed regarding when an individual is no longer infectious before Ct values alone can be used to inform decision making. Pertaining to the use of qualitative RT-PCR Ct values as a surrogate, *‘caution is advised when applying published correlations of Ct values with disease severity or as a predictor of active infection and transmissibility*,’ per the joint statement of the Infectious Diseases Society of America (IDSA) and the Association for Molecular Pathology (AMP) on the use of SARS-CoV-2 PCR Ct values for clinical decision-making.^21^ In select cases, adjunctive serological testing and subgenomic RNA testing may be considered on a case-by-case basis to circumvent the divorce between PCR and infectivity.

While the optimal dosing and timing for COVID-19 vaccination remain elusive in patients with cancer, all patients undergoing active treatment should be considered as ‘highly prioritized’. While patients who have received B-cell depleting agents in the preceding months may not mount an adequate humoral immune response with standard dosing regimen, patients undergoing immunotherapy are theoretically likely to develop protective immunity with the standard dosing. Longitudinal studies examining antibody and cellular immune responses, as well as randomized trials evaluating safety and efficacy of vaccine in patients undergoing ICIs, are needed. Universally, the risk of nosocomial transmission should be minimized by vaccinating all healthcare workers caring for patients with cancer, and strict mitigation strategies should remain in place across cancer centers. Expert opinion of a committee of the Society for Immunotherapy of Cancer (SITC) recommends that ‘*all cancer patients receiving approved or investigational immunotherapyas part of their treatment regimen, either as standard of care or as part ofclinical trials and without a general contraindication to vaccination, should/could receive an FDA approved and/or authorized SARS-CoV-2 vaccination whenmade available to them’* 22.

While there are no data, or any hypotheses that may be generated, on the differential efficacy and safety of different types of SARS-CoV-2 vaccines based on the differences in the manufacturing platforms/technology, any vaccine could hypothetically lead to an exaggerated immune response in ICI recipients. A non-randomized prospective study among ICI recipients, with 23 patients with lung cancer and 11 age-matched healthy controls, demonstrated a high rate of irAE (52%) occurring at a median time of 3 months after a trivalent inactivated influenza vaccine.^23^ However, the efficacy of the vaccine was not diminished as compared with healthy controls. Further larger-scale studies with control arms demonstrated similar efficacy without significant irAE.^4 24^ These data, coupled with ICI safety in other chronic viral infections, suggest that patients undergoing ICIs should mount an adequate immune response to COVID-19 vaccination. Further studies are urgently needed to evaluate toxicity and to elucidate the risk of irAE with COVID-19 vaccinations.

As oncologists continue to face tough decisions while prescribing life-prolonging immunotherapy in an era when the COVID-19 pandemic puts these profoundly immunosuppressed patients at substantial risk of irAEs, it is imperative that an informed and consensus decision is reached between the provider and the patient. Both parties ought to be aware of the risks that the immunotherapy regimen poses and should weigh that against the benefit ICIs will confer in terms of the intent of treatment.
